# Early and Late Endograft Limb Proximal Migration with Resulting Type 1b Endoleak following an EVAR for Ruptured AAA

**DOI:** 10.1155/2017/4931282

**Published:** 2017-01-31

**Authors:** Patrick T. Jasinski, Demetri Adrahtas, Spyridon Monastiriotis, Apostolos K. Tassiopoulos

**Affiliations:** Division of Vascular and Endovascular Surgery, Stony Brook Medicine, Stony Brook, NY, USA

## Abstract

*Introduction*. Seal zone failure after EVAR leads to type 1 endoleaks and increases the risk of delayed aortic rupture. Type 1b endoleaks, although rare, represent a true risk to the repair.* Case Presentation*. We report the case of a 65-year-old female who underwent emergent endovascular repair for a ruptured infrarenal abdominal aortic aneurysm and developed bilateral type 1b endoleaks following proximal migration of both endograft limbs. The right-side failure was diagnosed within 48 hours from the initial repair and the left side at the 1-year follow-up. Both sides were successfully treated with endovascular techniques. A review of the literature with an analysis of potential risk factors is also reported.* Conclusion*. For patients undergoing EVAR for ruptured AAA and with noncalcified iliac arteries, more aggressive oversizing of the iliac limbs is recommended to prevents distal seal zone failures.

## 1. Introduction

Seal zone integrity is a major determinant of the success rate of endovascular aortic aneurysm repair (EVAR). Several risk factors including neck dilatation, neck thrombus, calcifications endograft size mismatch, and endograft migration may contribute to the failure of the seal zone and subsequent type 1 endoleaks. Although less frequently described, distal seal zone failures and type 1b endoleaks represent a true threat for EVAR failure and post-EVAR aortic rupture.

## 2. Case Report

**Figure 1:**
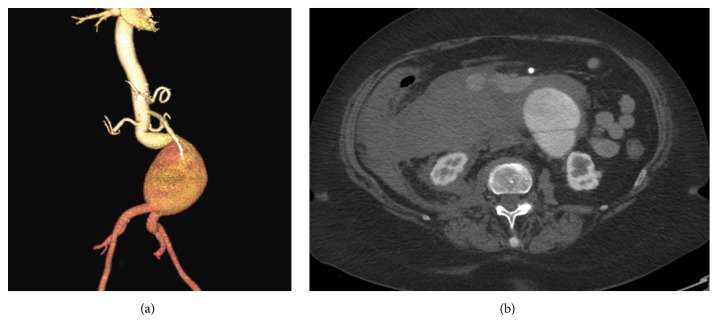
Computed tomography angiogram of ruptured AAA at initial presentation to the emergency department. (a) Three-dimensional reconstruction of the aneurysm sac. (b) Corresponding cross-sectional demonstrating rupture of AAA with active extravasation of contrast into the retroperitoneum.

A 65-year-old female with a known history of abdominal aortic aneurysm (AAA) and nephrolithiasis had avoided elective repair for years. She presented to the emergency department with severe abdominal and back pain and hypotension and was found on CT angiography to have a contained rupture of her infrarenal aneurysm that measured 7.9 cm in diameter and had significant proximal neck and iliac angulation (Figure 1).

**Figure 2:**
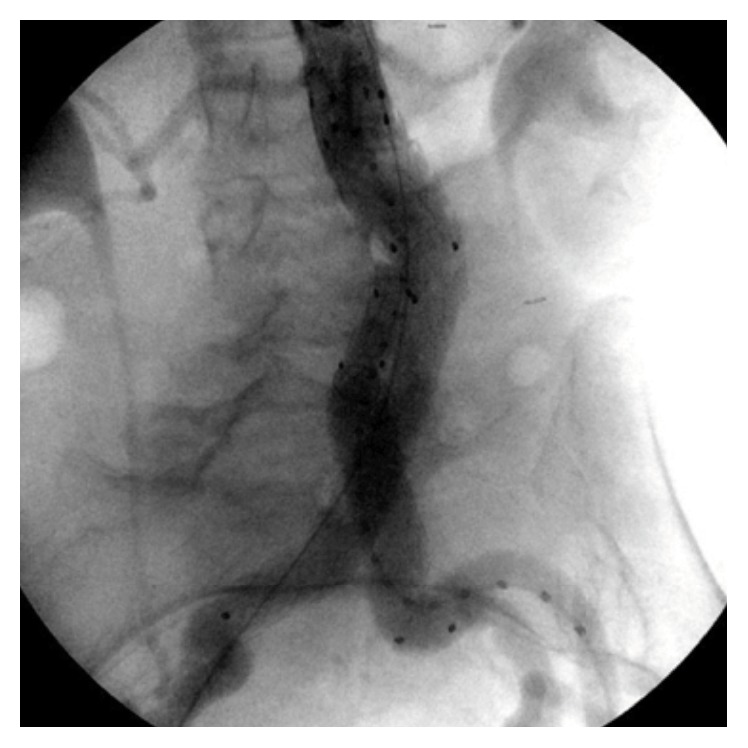
Intraoperative aortogram at the initial procedure showing the correct placement of the endograft and limbs bilaterally.

The anatomy was deemed suitable for EVAR (proximal aortic diameter 19 mm, aortic neck length 40 mm; LCIA: proximal 17 mm, distal 14 mm, length 42 mm; RCIA: proximal 12 mm, distal 14 mm, length 40 mm). Iliac tortuosity index was calculated demonstrating mild tortuosity on the left side and absent tortuosity on the right side (1.44 and 1.21, resp.) [1, 2]. Endovascular repair was carried out with a Medtronic Endurant® bifurcated endograft (23 × 16 × 166 mm) which was delivered via the right common femoral artery. The contralateral limb was deployed with its distal end landing approximately 1 cm from the left common iliac artery bifurcation. At the completion arteriogram, no endoleak was identified and the distal ends of the iliac limbs were confirmed with a centimeter within the bifurcation of the iliac arteries (Figure 2). The patient's hemodynamics normalized and she was transferred postoperatively to the ICU for monitoring.

**Figure 3:**
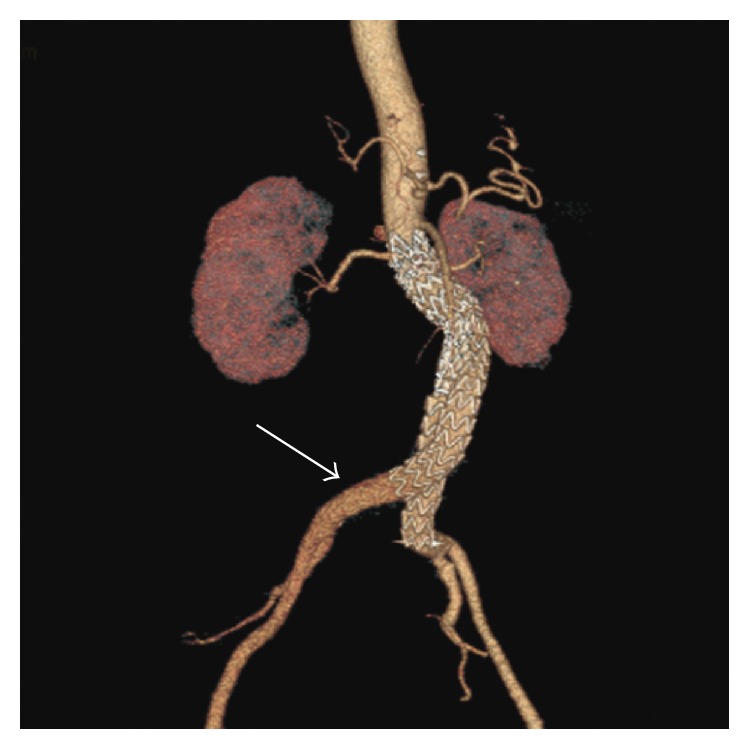
Computed tomography angiography performed on postoperative day 2 demonstrating the proximal migration of the right endograft limb (arrow).

**Figure 4:**
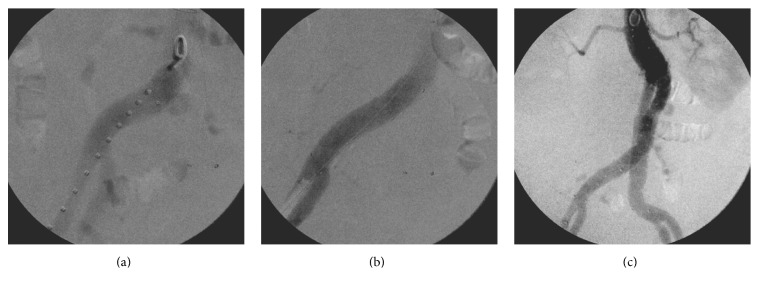
Intraoperative angiogram on postoperative day 2 demonstrating the acute proximal migration in the right common iliac artery resulting from the large aneurysm sac and the postimplantation change of the aortic anatomy.

On postoperative day 2, the patient became hypotensive again and was found to have clinical signs of ongoing hemorrhage. A repeat CTA was performed and concern for a type 1b endoleak at the distal end of the right common iliac artery limb was raised with high suspicion of active hemorrhage within the retroperitoneum. Further review of the CTA demonstrated proximal migration of the right limb with a significant decrease in the length of the seal zone (Figure 3). The patient was immediately transferred to the operating room and a retrograde arteriogram confirmed the right type 1b endoleak (Figure 4). The right endograft limb was extended distally to the right common iliac bifurcation with complete sealing of the endoleak. The patient was discharged a week later and follow-up CTs at 1 and 6 months showed regression of the aneurysm sac without evidence of type 1 or type 3 endoleaks. A small type 2 endoleak from a pair of lumbar arteries was seen.

**Figure 5:**
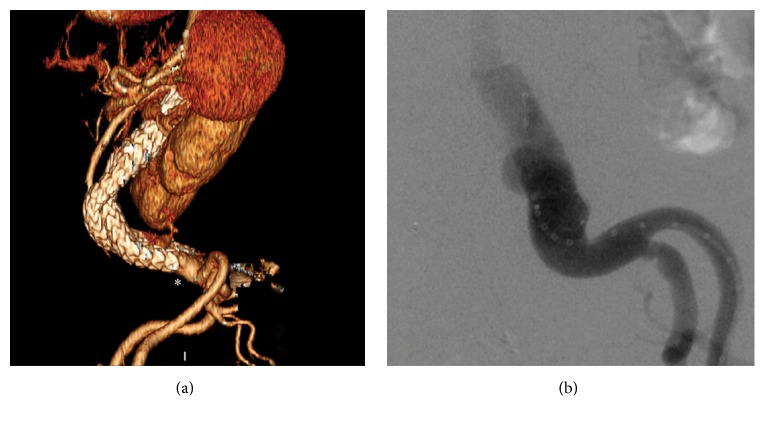
Follow-up at 12 months demonstrates a proximal migration of the left endograft limb (asterisk) on three-dimensional reconstruction of the CTA (a), leading to a type 1b endoleak as demonstrated in the aortogram (b).

**Figure 6:**
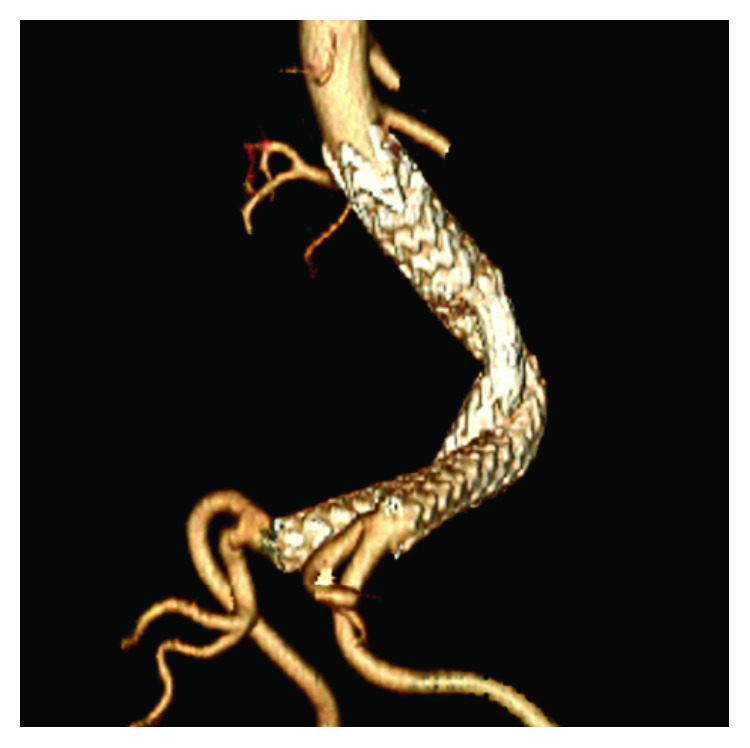
Surveillance follow-up CTA at 18 months. Three-dimensional reconstruction demonstrates the correct placement of the bilateral iliac limbs at the seal zones without evidence of endoleaks.

At the 12-month CT, however, enlargement of the aneurysm sac with suggestion of a new left type 1b endoleak and persistence of the type 2 endoleak was seen. Proximal migration of the left limb of the endograft was documented by comparison of the 12-month CTA images to the intraoperative arteriogram during the original repair and the subsequent CTA images at 1 and 6 months (Figure 5). The new type 1b endoleak was repaired by extension of the left endograft limb to the iliac bifurcation. A CT angiogram 6 months later confirmed complete resolution of the type 1b endoleak and a stable size of the aneurysm sac (Figure 6).

## 3. Discussion

EVAR is a widely used approach for treating abdominal aortic aneurysms with excellent perioperative outcomes in both elective and emergency procedures. However, long-term complications such as endoleaks, device migration, graft infection, and delayed rupture may threaten the initial success of the repair [3, 4]. Type 1 endoleaks result from failure of the seal zones at the proximal (Ia) or distal (Ib) attachment sites of the endograft and may be evident at the time of the repair or develop over time [5]. A number of risk factors for seal zone failure have been reported including size mismatch between the endograft and the aorta, the presence of mural thrombus or calcifications at the seal zones, and proximal neck challenges (short length, increased angulation, and reversed tapering) [5, 6]. When recognized, type 1 endoleaks should be repaired to avoid persistent exposure of the aneurysm sac to the systemic circulation and pressurization as this may lead to further growth and late rupture of an aneurysm [7, 8]. In recent years, less aggressive oversizing of the endograft limbs has been suggested to reduce the risk of limb occlusions and retrograde displacement as a result of the pulsatile flow [8, 9].

Migrations of the iliac limb extensions have been well described in the current literature [10]. In a prospective two-center analysis by Bisdas et al., the investigators examined the durability of the Endurant endograft in 273 all-comers. Device migration was reported in only 2 patients, leading to a type 1b endoleak at 49 and 53 months as a result of iliac artery aneurysmal progression [11]. Zandvoort et al. studied outcomes following EVAR in 100 consecutive patients and published their 4-year results. Although no graft migration was reported, treatment for type 1b endoleak was necessary for 5 patients and resulted from worsening of iliac aneurysmal disease [12]. Two other studies have reported excellent results with regard to type 1 endoleaks using the Medtronic system [13, 14]. Although type 1 endoleaks were significantly higher with challenging neck anatomy at 30 days, no significant difference was reported at 1 year.

Our patient was treated expeditiously due to her acute presentation. She had a large aneurysm sac with relatively short and angulated common iliac arteries (Figure 1). She developed early limb migration on the right side and a type 1b endoleak within 48 hours despite appropriate landing of the endograft limb during the initial procedure as seen in Figure 2. We measured a proximal migration of at least 2.5 cm on that side within 48 hours from the repair. The diameter of the endograft limb that we utilized was oversized by 1.8 mm compared to the diameter of the common iliac arteries (common iliac artery diameter was 14.2 mm bilaterally; endograft limb diameter was 16 mm at the original repair). When measuring the diameter of the iliac arteries at 6 months after the intervention, the vessel diameter was slightly higher measuring 1.56 cm on the right and 1.54 cm on the left side. We speculate that this discrepancy is likely due to the fact that the original CT scan images were obtained while the patient was hypotensive as a result of the rupture and deliberately kept underresuscitated to avoid further blood loss prior to the repair. We believe that this led us to underestimate the true diameter of the iliac arteries and only minimally oversize the endograft limbs. The usual practice is to oversize the endograft by 10 to 15% at the proximal seal zone and 1 to 4 mm at the distal seal zone. We believe that the true diameter of the iliac arteries was underestimated in this patient as a result of vasoconstriction leading to the early migration of the inadequately oversized iliac limbs.

**Figure 7:**
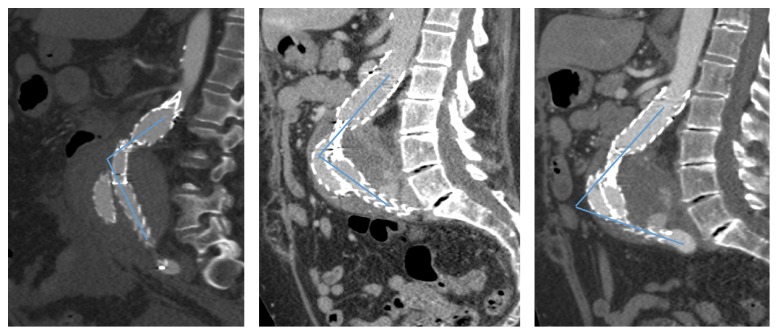
Sagittal view of computed tomography performed at 1, 6, and 12 months of follow-up demonstrating the anterior displacement of the aortic endograft.

In addition, over the course of the first year after initial implantation, serial CT scans demonstrate a progressive anterior displacement of the endograft up to the point where the endograft limbs reach the anterior aneurysm wall (Figure 7). Although this anterior displacement clearly contributed to the proximal migration of the endograft, we assume that the minimal oversizing of the endograft limbs comparing to the true diameter of the iliac arteries was also a significant factor predisposing to proximal limb migration.

## 4. Conclusion

We suggest that in patients with ruptured AAA and noncalcified iliac arteries who are considered for endovascular repair more aggressive oversizing of the iliac limbs could potentially prevent distal seal zone failures. This report also underscores the need for ongoing aneurysm surveillance in these patients as aortic remodeling can contribute to endograft-related complications.
